# Association between muscle strength and depressive symptoms among Chinese female college freshmen: a cross-sectional study

**DOI:** 10.1186/s12891-020-03478-w

**Published:** 2020-07-31

**Authors:** Zhongyu Ren, Jianhua Cao, Yingke Li, Peng Cheng, Bing Cao, Zongji Hao, Hui Yao, Dongzhe Shi, Bin Liu, Chang Chen, Guang Yang, Li Peng, Liya Guo

**Affiliations:** 1 College of Physical Education and Key Laboratory of Physical Fitness Evaluation & Motor Function Monitoring, General Administration of Sport of China, Institute of Sports Science, Southwest University, Chongqing, 400715 China; 2 College of Physical Education, Chongqing Nursing Vocational College, Chongqing, 402763 China; 3 Key Laboratory of Cognition and Personality, Faculty of Psychology, Ministry of Education, Southwest University, Chongqing, 400715 China; 4 College of Physical Education, Xinhua College of Sun Yat-Sen University, Guangzhou, 523133 China; 5 School of Physical Education, and Chinese Center of Exercise Epidemiology, Northeast Normal University, Changchun, 130024 China

**Keywords:** Depressive symptoms, Muscular strength, Chinese college freshman, Handgrip strength

## Abstract

**Background:**

Increased physical activity level is related to lower risk of depressive symptoms, and there is an inverse association between muscle strength and risk of depressive symptoms among the elderly. Although there is evidence of an inverse association between muscle strength and depressive symptoms, the relationship between these variables in a younger population is still unknown. This study aimed to examine the association between handgrip strength, a representative indicator of skeletal muscle strength, and the risk of depressive symptoms among Chinese female college freshmen.

**Methods:**

A cross-sectional study was conducted among 867 participants aged between 16 and 23 years. Handgrip strength was measured with a handheld digital Smedley dynamometer, and handgrip strength relative to body weight (kg/kg) was calculated and was classified into tertiles as follows: low (0.32–0.50), medium (0.51–0.58), and high (0.59–0.94). Depressive symptoms were evaluated using the 20-item Zung self-rating depression scale (SDS), and three cutoff points were used to indicate different depression levels.

**Results:**

We found that 10.7% of participants were classified as having severe depressive symptoms using an SDS score of 50 as the cutoff point. After adjusting for potential confounders, the adjusted odds ratios and 95% confidence intervals (CIs) for moderate-to-severe depressive symptoms across tertiles of the relative handgrip strength were 1.00 (reference) for tertile 1, 0.614 (0.353, 1.069) for tertile 2, and 0.537 (0.292, 0.988) for tertile 3 (P for trend = 0.041). The significant associations remained when other cutoff points (SDS scores: 48 or 45) were used. Interactions between handgrip strength and potential confounders for depressive symptoms in the final models were not significant.

**Conclusions:**

Our findings indicate that handgrip strength is inversely and independently related to the risk of depressive symptoms among Chinese female college freshmen. The present findings can help develop an effective intervention strategy against depression. Further intervention studies are needed to explore the mechanisms underlying the effects of handgrip strength on depressive symptoms.

## Background

Attending university for the first time is an important stage of life for college students, and they are required to accomplish academic tasks and negotiate a new social world due to unfamiliarity of university life. Particularly, first-year students may struggle more with issues such as their transition and adjustment to college life, and many of them may have to face academic and life stresses in today’s competitive world. Accumulating studies have indicated that many college students experience psychological disturbances, such as depression. For example, approximately 53% of freshmen reported having experienced depression since beginning college in the United States [1]; 20–30% of college freshmen reported having major depression in Japan [2]; similarly, the prevalence rates of depression or depressive symptoms among first-year university students were 24.8 and 43.9% in mainland China and Hong Kong, respectively [3]. The prevalence rate of depressive symptoms among college freshmen in these studies differs from that among the general population (37.9% for depressive symptoms) [4], probably due to the use of different measurement tools, different methodologies, and different appraisal standards. Notably, depression contributes to the onset of cancer [5], diabetes mellitus [6], coronary heart disease [6], stroke [7], and metabolic syndrome [8], and at its worst, it can lead to suicide [9]. Thus, identification of potentially modifiable risk factors for depression is imperative to the development of an effective preventive strategy.

Although increasing levels of physical activity (PA) could protect against developing depression [10], only a few studies have examined objective measurements of physical fitness in relation to depression. Physical fitness, including aerobic power, joint flexibility, muscle strength, and endurance [11], may be associated with depression because improved physical fitness represents an adaptation to regular PA [12]. In addition, an inverse association between cardiorespiratory fitness and depression risk has been well-established among young [13] and middle-aged adults [14, 15]. Similarly, previous studies also reported an association between muscle strength and depression risk among the elderly population [16–18]. However, to our knowledge, no studies have investigated whether muscle strength has a significant association with depression at younger ages, and elucidation of this association may facilitate earlier and more effective preventive interventions for depression.

Handgrip strength is a simple, accurate, and quick measurement of general muscle strength [19]. This cross-sectional study aimed to investigate the association between relative handgrip strength and the risk of depressive symptoms in Chinese female college freshmen.

## Methods

### Participants

The Chongqing Nursing Vocational College Physical Fitness and Health (CNVCPFH) study is an ongoing prospective cohort study to assess the association between physical fitness and the health status of college students in Chongqing, China. The detailed study design has been previously published [20].

We conducted a cross-sectional analysis using baseline data from the CNVCPFH study in this study. A total of 1094 college freshmen were recruited in the first week of October 2018 (baseline period) through advertisements or from referrals, and all of them agreed to participate in this study. Written informed consent was obtained from participants aged ≥16 years or their parents or legal guardians for participants aged < 16 years. Participants who did not complete the handgrip strength assessment (*n* = 59), as well as those with missing information on age, height and/or bodyweight measurements, and PA (*n* = 62), were excluded from this study. Because the number of male participants (*n* = 106) was too small to perform a multiple logistic regression analysis, male participants were therefore excluded from the study. Finally, 867 female participants aged between 16 and 23 years (mean age, 18.7 years; SD, 1.0) were included in this study. Ethics approval was obtained from the Ethical Committee of the College of Physical Education of Southwest University.

### Assessment of handgrip strength

Handgrip strength was assessed using a dynamometer (EH101; CAMRY, Guangdong, China). All participants were asked to adjust the dynamometer width for optimal hand comfort and to relax their arm in a standing and stationary position. Each participant made four attempts using each hand with a brief interval between trials. The highest weight in kilograms of all handgrip strength measurements was used as a representative value of muscle strength. Because of skewed distributions, handgrip strength relative to body weight (kg/kg) was calculated and was classified into tertiles as follows: low (0.32–0.50), medium (0.51–0.58), and high (0.59–0.94).

### Assessment of depressive symptoms

The Zung self-rating depression scale (SDS) was used to examine depression severity [21]. It comprises of 20 items, and each item score ranges from 1 to 4, with a sum score between 20 and 80. Higher scores represent a more severe depressive state, and scores higher than 50 clinically reflect depressive symptoms [21]. In order to increase the sensitivity of the detection and distinguish the severity of depressive symptoms, we used two cutoff points (45 and 48) to distinguish the mild depression levels [22, 23]. The reliability and validity of the Chinese version of SDS have been described in a previous study [24]. In the present study, the Cronbach’s α coefficient for the scale was 0.788, indicating that the Chinese version of the SDS in this study has a strong internal consistency.

### Relevant covariates

The anthropometric variables (height and body weight) were measured using a standard protocol. Demographic variables included age (continuous variable), only one child (yes or no), race (Han nationality, Tujia nationality, Miao nationality, or others), father’s educational level (senior high school or below, college, or postgraduate), mother’s educational level (senior high school or below, college, or postgraduate), and parent’s marital status (married, widowed, or divorced). Lifestyle factors including smoking status (never, occasionally, or regularly), drinking status (never, occasionally, or regularly), sleep duration (6-8 h/d or <6 and >8 h/d), and sleep quality (good or not) were assessed using a self-administered questionnaire. Levels of PA were assessed by the International Physical Activity Questionnaire (IPAQ) (short version) [25]. Total weekly PA was calculated as metabolic equivalents × hour/week [25] and was classified into tertiles.

### Data analysis

Continuous and categorical variables are presented as geometric least square mean (95% CI) or proportions (95% CI). Non-normal continuous variables were log-transformed for multivariate statistical analyses. For analyses of participants’ characteristics, continuous and categorical variables were compared using analysis of variance (ANOVA).

Depressive symptoms were used as dependent variables, and relative handgrip strength was classified as independent variables. Multiple logistic regression analysis was employed to examine the relationship between relative handgrip strength and depressive symptoms. Model 1 was a crude univariate model; model 2 was adjusted for age (continuous variable), BMI (continuous variable); and model 3 was further adjusted for race (Han nationality, Tujia nationality, Miao nationality, and other nationalities), only one child (yes or no), father education level (senior high school or below, college, and postgraduate), mother education (senior high school or below, college, and postgraduate), smoking status (never, occasionally, or regularly), drinking status (never, occasionally, or regularly), physical activity level (low, middle, and high level), sleep quality (good or not), sleep duration (6-8 h/d or <6 and >8 h/d), and parent’s marital status (married, widowed, and divorced). Interactions between handgrip strength levels and confounders of depressive symptoms were tested by the addition of cross-product terms to the regression model. *P*-values < 0.05 were considered statistically significant for all two-sided tests. All tests were performed using the IBM SPSS Statistics 24.0 software (IBM SPSS Inc., Chicago, IL, USA).

## Results

Participants’ characteristics according to the relative handgrip strength are presented in Table 1. The BMI differed significantly among tertiles of the relative handgrip strength (P for trend: < 0.001). Total scores of depressive symptoms differed significantly among tertiles of the relative handgrip strength (P for trends = 0.014). In addition, no significant differences in other variables were observed among the tertiles of the relative handgrip strength.

**Table 1:** Participants’ characteristics according to tertiles of relative handgrip strength^a^

	Tertile 1 (low) (*n* = 286)	Tertile 2 (*n* = 289)	Tertile 3 (high) (n = 292)	P for trend^b^
Mean (kg/kg)	0.45	0.54	0.64	–
Age, years	18.6 (18.5, 18.7)	18.6 (18.5, 18.7)	18.7 (18.6, 18.8)	0.278
BMI, Kg/m^2^	21.6 (21.3, 21.9)	20.0 (19.7, 20.2)	19.0 (18.8, 19.2)	< 0.001
Smoking status, %				
Occasionally	5.6 (2.9, 8.3)	5.2 (2.6, 7.8)	3.4 (1.3, 5.5)	0.425
Regularly	0.3 (−0.3, 1.0)	0.3 (−0.3, 1.0)	0.3 (−0.3, 1.0)	1.000
Drinking status, %				
Occasionally	55.9 (50.2, 61.7)	46.4 (40.6, 52.2)	48.6 (42.9, 54.4)	0.056
Regularly	0.0 (0.0, 0.0)	1.7 (0.2, 3.2)	1.4 (0.0, 2.7)	0.097
Tertile of physical activity, %				
Middle (range 28.43–58.50 MET·h·week-1)	32.2 (26.7, 37.6)	34.3 (28.8, 39.8)	33.6 (28.1, 39.0)	0.864
High (range>58.50 MET·h·week-1)	36.0 (30.4, 41.6)	31.1 (25.8, 36.5)	33.2 (27.8, 38.7)	0.463
Sleep duration (6–8 h), %	88.0 (84.0, 92.0)	91.0 (88.0, 94.0)	90.0 (87.0, 94.0)	0.480
Sleep quality (good), %	75.2 (70.1, 80.2)	74.7 (69.7, 79.8)	77.1 (72.2, 81.9)	0.788
Race, %				
Han nationality	86.0 (82.0, 90.1)	87.2 (83.3, 91.1)	89.4 (85.8, 92.9)	0.461
Tujia nationality	9.4 (6.0, 12.9)	7.3 (4.3, 10.3)	6.5 (3.7, 9.4)	0.393
Miao nationality	2.8 (0.9, 4.7)	3.5 (1.3, 5.6)	1.7 (0.2, 3.2)	0.417
Father education level, %				
senior high school or below	93.4 (90.5, 96.3)	92.0 (88.9, 95.2)	92.8 (89.8, 95.8)	0.831
college	6.3 (3.5, 9.1)	8.0 (4.8, 11.1)	6.9 (3.9, 9.8)	0.730
Mother education level, %				
Senior high school or below	95.5 (93.0, 97.9)	95.2 (92.7, 97.7)	96.6 (94.5, 98.7)	0.672
College	4.6 (2.1, 7.0)	4.8 (2.4, 7.3)	3.4 (1.3, 5.5)	0.672
Parent’s marital status, %				
Widowed	3.9 (1.6, 6.1)	4.8 (2.4, 7.3)	2.4 (0.6, 4.2)	0.291
Divorced	14.7 (10.6, 18.8)	13.5 (9.5, 17.5)	10.3 (6.8, 13.8)	0.259
Only one child, %	27.6 (22.4, 32.8)	26.6 (21.5, 31.8)	21.6 (16.8, 26.3)	0.199
SDS scores	40.2 (39.3, 41.1)	39.6 (38.7, 40.4)	38.6 (37.8, 39.5)	0.014

*PA* physical activity, *BMI* body mass index, *MET* metabolic equivalent, *SDS* self-rating depression scale

^a^Continuous variable without a normal distribution were log-transformed; Continuous variables are expressed as estimated geometric means (95%CI) and categorical variables are expressed as percentages (95%CI)

^b^Linear trends were assessed using analysis of variance for continuous variables and categorical variables

We further found that 10.7% of freshmen were classified as having severe depressive symptoms using an SDS score of 50 as the cutoff point. Table 2 shows the crude and adjusted significant relationships between the tertiles of relative handgrip strength and the risk of severe depressive symptoms. In the final model, the adjusted ORs for severe depressive symptoms across tertiles of relative handgrip strength were 1.00 (reference) for tertile 1, 0.614 (95% CI: 0.353, 1.069) for tertile 2, and 0.537 (95% CI: 0.292, 0.988) for tertile 3 (P for trend: 0.041). Similar relationships were observed when an SDS score of 45 or 48 was used as the cutoff point [see eTables S1 and S2]. Tests conducted to identify interactions between the tertile of relative handgrip strength and potential confounders for depressive symptoms in the final models were not found to be significant.

**Table 2:** Adjusted odds ratios (95% confidence interval) of associations of relative handgrip strength with depressive symptoms (SDS ≥ 50) among Chinese female college students

*N* = 867	Number of Case	Model 1 ^a^	Model 2 ^b^	Model 3 ^c^
Tertile 1 (n = 286)	42	1.000 (reference) ^d^	1.000 (reference)	1.000 (reference)
Tertile 2 (*n* = 289)	28	0.623 (0.375, 1.037)	0.629 (0.371, 1.068)	0.614 (0.353, 1.069)
Tertile 3 (*n* = 292)	23	0.497 (0.290, 0.850)	0.508 (0.283, 0.913)	0.537 (0.292, 0.988)
P for trend ^e^	–	0.009	0.021	0.041

^a^Model 1 are a crude univariate model;

^b^Model 2 adjusted for age (continuous variable), BMI (continuous variable);

^c^ Model 3 additionally adjusted for race (han nationality, tujia nationality, miao nationality and other nationality), only one child (yes or no), father education (senior high school or below, college and postgraduate), mother education (senior high school or below, college and postgraduate), smoking status (never, occasionally, or regularly), drinking status (never, occasionally, or regularly), physical activity level (low, middle, and high), sleep quality (good or not), sleep duration (6-8 h/d or <6 and >8 h/d) and parent’s marital status (married, widowed and divorced)

^d^Adjusted data are expressed as odds ratio (95% confidence intervals)

^e^p for trend were obtained using multivariate logistic regression analyses

## Discussion

The relationship between higher handgrip strength (an indicator of overall muscle strength) and lower risk of depressive symptoms has been widely established [16–18]. However, this relationship has not been confirmed among younger adults. This cross-sectional study was conducted among Chinese female college freshmen to assess the relationship between handgrip strength and depressive symptoms. Multivariate logistic analyses showed that higher handgrip strength was significantly and independently associated with a lower risk of depressive symptoms after adjustment for potential confounders. Our study expands on previous findings by demonstrating that increased handgrip strength could be independently related to low risk of depressive symptoms among Chinese female college freshmen.

Depressive symptoms are chronic and debilitating mood disorders, and the exact etiology for depressive symptoms is unknown. PA levels could mediate the inverse association between handgrip strength and depressive symptoms, and primary monoamines in the brain, including noradrenaline, dopamine, and serotonin, play important roles in arousal and attention and are involved in the pathogenesis of depression [26, 30]. Interestingly, the anti-depressive potential of PA might be associated with increased levels of primary monoamines. Indeed, accumulating evidence suggests that physical exercise exerts a positive effect on various neurotransmitter systems, which regulate levels of serotonin [27], dopamine [28], and noradrenaline [29]; notably, these neurotransmitters are closely related to onset and development of depressive symptoms. In addition to physiological state, physical activity is also beneficial to one’s psychological status. Regularly participating in physical activity can usually distract individuals from unpleasant stimuli, thereby improving depression [30]. The significant relationship between handgrip strength and depressive symptoms remained after adjustment for PA level analyzed by the short version of IPAQ, and further studies are needed to investigate the effect of other confounding PA types on the association of handgrip strength with depressive symptoms [25, 31].

Activation of skeletal muscle increases secretion of peroxisome proliferator-activated receptor-gamma co-activator-1 alpha 1 [32], which promotes skeletal muscle expression of kynurenine aminotransferase, thereby facilitating transformation of kynurenine into kynurenic acid [33], a metabolism product unable to cross the blood-brain barrier [34]. To alleviate stress-induced depression, it is essential to prevent kynurenine from crossing the blood-brain barrier to disrupt neural plasticity [32]. However, plasma kynurenine levels were not examined in this study. Therefore, future studies are needed to determine whether plasma kynurenine levels mediate this relationship between handgrip strength and the risk of depressive symptoms.

The association between the incidence of depressive symptoms and the decline in skeletal muscle strength in Chinese college students is well known [35, 36]. This present study further confirmed the association between handgrip strength and depressive symptoms in Chinese female college freshmen. Considering the complex etiology of depressive symptoms, the analyses in this population-based study were adjusted for a considerable number of confounding factors. Accumulating studies have demonstrated significant relationships of depressive symptoms with race [37], father’s educational level [38], mother’s educational level [38], parent’s marital status [38], and being the only one child [39]; notably, these socio-demographic variables are also associated with physical fitness [40–42]. However, the adjustment of these socio-demographic variables did not have an effect on the significant association between handgrip strength and depressive symptoms. In addition, the present analyses were also adjusted for BMI [43], smoking status [43], drinking status [44], sleep duration [45, 46], and sleep quality [47, 48], and the significant association between handgrip strength and depressive symptoms still remained. The findings suggest that handgrip strength is independently associated with depressive symptoms.

### Implications

The current study further strengthens the evidence that greater muscle strength could be associated with a lower risk of depressive symptoms among the younger population, e.g., college students. In clinical settings, physicians or health care professionals often suggest their clients to improve or maintain higher physical activity levels to reduce the risk of depression. This evidence-based study suggests that handgrip strength, which objectively represents the physical activity level, should be paid more attention to reduce the risk of depression because it could be readily and accurately measured compared with physical activity.

### Limitations

There are several limitations to our study. First, although this study provides some insights into the potential mechanisms by which higher handgrip strength level is related to lower risk of depressive symptoms, the causation between handgrip strength and depressive symptoms could not be established due to the cross-sectional study design. In addition, a longitudinal studies-based review indicates that baseline depression may be a significant risk factor for the development of sedentary lifestyle or decreased level of physical activity [49], probably because individuals with depressive symptoms have lower confidence and self-esteem, elevated feelings of self-criticism, and excessive inappropriate guilt; these negative forms of motivation prevent individuals to participate in active and healthy behaviors [50] and therefore may have negative effects on engaging in physical activity in young adults [51]. Thus, there may be bidirectional associations between muscle strength and depressive symptoms in young adults. Future prospective cohort or intervention studies are required to better elucidate the relationships between handgrip strength and the risk of depressive symptoms. Second, although three cutoff points (SDS scores: 45, 48, and 50) were used to define moderate-to-severe depressive symptoms, we were unable to establish a clinical diagnosis to determine the exact morbidity rate associated with depression. Third, in the current study, the participants were limited to Chinese female college freshmen, and whether the abovementioned relationship also exists in Chinese male college freshmen, other regional college students, or college students at other grades remains unknown. Therefore, further studies with a larger sample of representative Chinese college students are necessary to confirm our present findings. Fourth, physical fitness is generally composed of aerobic power, joint flexibility, muscle strength, and endurance [11]. However, we did not investigate the influence of all these physical fitness components on depressive symptoms. Because higher cardiorespiratory fitness (aerobic power) level has a significant association with decreased incidence of depressive symptoms [52], the significant relationship of handgrip strength with the risk of depressive symptoms might be attenuated when adjusting for cardiorespiratory fitness. In addition, accumulating studies indicate that physical activity is associated with muscle strength among Chinese college students [53], and physical activity level is related to muscle strength and aerobic power, but not joint flexibility and muscle endurance, in Chinese adolescents [54]. Taken together, muscle strength, but not other physical fitness components, is inversely related to the risk of depressive symptoms.

## Conclusions

This cross-sectional study assessed the relationship between handgrip strength and depressive symptoms in Chinese female college freshmen. We demonstrated that increased handgrip strength was significantly and independently related to a lower risk of depressive symptoms. The present findings can help develop an effective intervention strategy against depression. Further intervention studies are needed to explore the causality between handgrip strength and the risk of depressive symptoms.

## Supplementary information

**Additional file 1: Table S1.** Adjusted odds ratios (95% confidence interval) of associations of relative handgrip strength with depressive symptoms (SDS ≥45) among Chinese female college students. **Table S2.** Adjusted odds ratios (95% confidence interval) of associations of relative handgrip strength with depressive symptoms (SDS ≥48) among Chinese female college students.

## Data Availability

The datasets used and/or analyzed during the current study are available from the corresponding author on reasonable request.

## Abbreviations

ANOVA: Analysis of variance
BMI: Body mass index
CNVCPFH: Chongqing Nursing Vocational College Physical Fitness and Health
PA: Physical activity
IPAQ: International Physical Activity Questionnaire
SDS: Zung self-rating depression scale
