# Supplementary material for: Association between muscle strength and depressive symptoms among Chinese female college freshmen: a cross-sectional study
Source: BMC Musculoskelet Disord. 2020 Jul 31;21:510. doi: 10.1186/s12891-020-03478-w (PMC7395416; doi:10.1186/s12891-020-03478-w)
Supplement: Supplementary file 1 — Additional file 1: Table S1. Adjusted odds ratios (95% confidence interval) of associations of relative handgrip strength with depressive symptoms (SDS ≥45) among Chinese female college students. Table S2. Adjusted odds ratios (95% confidence interval) of associations of relative handgrip strength with depressive symptoms (SDS ≥48) among Chinese female college students. [file 12891_2020_3478_MOESM1_ESM.docx]

**Table S1. Adjusted odds ratios (95% confidence interval) of associations of relative handgrip strength with depressive symptoms (SDS ≥45) among Chinese female college students.**

| N = 867 | Number of Case | Model 1 ^a^ | Model 2 ^b^ | Model 3 ^c^ |
| --- | --- | --- | --- | --- |
| Tertile 1 (n =286) | 42 | 1.000 (reference) ^d^ | 1.000 (reference) | 1.000 (reference) |
| Tertile 2 (n =289) | 28 | 0.784 (0.550, 1.118) | 0.735 (0.508, 1.063) | 0.721 (0.490, 1.059) |
| Tertile 3 (n =292) | 23 | 0.684 (0.478, 0.980) | 0.618 (0.416, 0.918) | 0.634 (0.420, 0.956) |
| P for trend ^e^ | — | 0.038 | 0.017 | 0.030 |

^a^ Model 1 are a crude univariate model;

^b^ Model 2 adjusted for age (continuous variable), BMI (continuous variable);

^c^ Model 3 additionally adjusted for race ( han nationality, tujia nationality, miao nationality and other nationality), only one child (yes or no), father education level (senior high school or less, college and postgraduate), mother education level (senior high school or less, college and postgraduate), smoking status (never, occasionally, or regularly), drinking status (never, occasionally, or regularly), physical activity level (low, middle, and high), sleep quality (good or not), sleep duration (6-8 h/d or ＜6 and ＞8 h/d) and parent's marital status (married, widowed and divorced)

^d^ Adjusted data are expressed as odds ratio (95% confidence intervals).

^e^ p for trend were obtained using multivariate logistic regression analyses.

**Table S2. Adjusted odds ratios (95% confidence interval) of associations of relative handgrip strength with depressive symptoms (SDS ≥48) among Chinese female college students.**

| N =867 | Number of Case | Model 1 ^a^ | Model 2 ^b^ | Model 3 ^c^ |
| --- | --- | --- | --- | --- |
| Tertile 1 (n =286) | 42 | 1.000 (reference) ^d^ | 1.000 (reference) | 1.000 (reference) |
| Tertile 2 (n =289) | 28 | 0.759 (0.502, 1.145) | 0.709 (0.461, 1.089) | 0.688 (0.437, 1.082) |
| Tertile 3 (n =292) | 23 | 0.482 (0.307, 0.756) | 0.436 (0.267, 0.712) | 0.440 (0.264, 0.735) |
| P for trend ^e^ | — | 0.001 | 0.001 | 0.002 |

^a^ Model 1 are a crude univariate model;

^b^ Model 2 adjusted for age (continuous variable), BMI (continuous variable);

^c^ Model 3 additionally adjusted for race ( han nationality, tujia nationality, miao nationality and other nationality), only one child (yes or no), father education level (senior high school or less, college and postgraduate), mother education level (senior high school or less, college and postgraduate), smoking status (never, occasionally, or regularly), drinking status (never, occasionally, or regularly), physical activity level (low, middle, and high), sleep quality (good or not), sleep duration (6-8 h/d or ＜6 and ＞8 h/d) and parent's marital status (married, widowed and divorced)

^d^ Adjusted data are expressed as odds ratio (95% confidence intervals).

^e^ p for trend were obtained using multivariate logistic regression analyses.
